# An integrative counselling program to promote active ageing for older people in Thai nursing homes: an intervention mixed methods design

**DOI:** 10.1186/s40359-021-00519-4

**Published:** 2021-01-26

**Authors:** Jantana Juthavantana, Nanchatsan Sakunpong, Ujsara Prasertsin, Monthira Charupheng, Sheibon Hassakama Lau

**Affiliations:** 1 Behaviour Science Research Institute, Srinakharinwirot University, 114 Sukhumvit 23, Klongtoeinuea, Wattana District, Bangkok, 10110 Thailand; 2 Educational and Psychological Test Bureau, Srinakharinwirot University, Bangkok, Thailand; 3 Faculty of Education, Srinakharinwirot University, Bangkok, Thailand; 4 School of Medicine, Medical Sciences and Nutrition, University of Aberdeen, Aberdeen, UK

**Keywords:** Active ageing, Counselling, Mixed methods research, Satir

## Abstract

**Background:**

Active ageing has been a rapidly developing field of study in light of the growing population of older people. Acknowledgement of the lack of a counselling program to promote active ageing for the older people in nursing homes led to the development of this study which aims to investigate active ageing of the Thai elderly in a nursing home in addition to promoting active ageing for them through integrative counselling.

**Methods:**

The study was conducted in a nursing home in Samut Prakan province, Thailand. The integrative counselling program referred to appropriate literature along with implementation of the Satir Model and Motivational Interviewing techniques. An intervention mixed methods design was applied in the study, which consisted of two phases. Phase 1 involved an investigation of the concept of active ageing, based on the context of older people in nursing homes by way of in-depth interviews, involving 5 participants. Phase 2 comprised of an investigation of the effects of an integrative counselling program to promote active ageing for older people in the same nursing home. There was a total of 16 participants in phase 2 which were divided equally into experimental and control groups respectively.

**Results:**

Phase 1 of the study showcased qualitative results of the progress of active ageing development in older people that resulted in 4 sub-themes (Health development, spiritual development, active engagement and psychosocial support). Two parameters were used to analyze the results in phase 2. The quantitative results showed that the active ageing score of participants in the experimental arm increased significantly after enrollment (*p* < 0.05). Furthermore, the experimental group had a higher overall active ageing score in comparison to the control group. Qualitative results of phase 2 elicited factors promoting active ageing in the elderly which included activities, group facilitator and group atmosphere. Both quantitative and qualitative results of phase 2 proved to be significant, showing that the program managed to develop active ageing in participants.

**Conclusion:**

Psychologists and multidisciplinary teams looking after older people in nursing homes are able to use this integrative counselling program for development of active ageing in the elderly population.

## Background

In the past, the notion of the “Thai family” typically involved an extended family involving different generations. Seniority and family bonding are highly regarded and were established through traditional Thai culture that was passed on through generations. That concept is somewhat different from the modern Thai family with more contemporary lifestyles and values. Indirectly, this has caused an increase in admission of older people into nursing homes in Thailand, namely due to declines in their health as well as the loss of independence in an absence of next of kin or the inability of other direct family members to provide adequate care for them [1].

By 2021, Thailand will become a “completely-aged society”, this refers to having more than 20% of the Thai population aged 60 years or above, or having more than 14% of said population aged 65 years or above, leading to the number of dependent older people reaching 13 million [2]. With this rapid increase, the number of older people admitted into nursing homes is expected to increase tremendously, bearing in mind that according to [3] the residents’ quality of life is lower than that of older people living in the community. Resulting in a rapid change to their psycho-physical balance, with a study conducted in Italy by Scocco, Rapattoni and Fantoni [1] concluding that admissions into nursing homes had done more harm than good to their cognitive functions and psychological state etc. The results proved to be similar in Thailand based on a pre-research visit conducted by the researcher to the studied nursing home, with findings that older people often lived a monotonous life without visits from relatives and with a lack of vitality towards life [4].

Even with the proven negative effects of a nursing home on the elderly population’s well-being, there is still no study undertaken on the potential application of counselling psychology to empower older people. For that reason, the researcher designed an integrative counselling program to address this matter. As stated by Carmen [5], ‘… one of the major problems specific to institutionalized elderly is depression and the feeling of isolation.’ emphasizing the importance of counselling as institutionalized elderly are more likely to seek for counselling as a result of feeling this way. Furthermore, Hill & Brettle’s [6] systematic review exhibited strong evidence supporting the efficacy of counselling in older people, particularly in treating anxiety, depression and in improvement of subjective well-being. Another study by [7] has shown that integrative group counselling was able to aid elders in Thailand to achieve successful ageing.

Most counsellors and psychologists prefer to integrate counselling and psychotherapy in practice. This is due to the better suitability for application in a real-life setting compared to standard approaches as integrative counselling combines different elements of specific therapies which allows for further customization to cater for the study population [8–10]. Integrative counselling is able to combine the different strengths from different psychological theories, effectively implementing the intervention intended into the studied population. Assimilative integration was used in the counselling program to incorporate both technical eclecticism and theoretical integration [11, 12]. This study relies on the Satir Model [13] for participants’ realisation of their self-worth and improving participant’s self-esteem in addition to Motivational Interviewing techniques [14] which encourages change in participants’ behaviour.

The integrative counselling program in this study was developed through the assimilation integrative approach based on the Satir Model [13]. Due to the Buddhist nature of the nursing home, the program designed for them adapted the Satir Model towards Buddhism. In general, the program aimed to promote insight, inner harmony, and self-esteem for older people. As a result, it was expected that the elderly would be better able to cope with challenges, have positive attitudes towards themselves and others, be able to accept their current reality and adjust their expectations towards themselves and others, be able to adjust well to the surrounding environment and situations, and have improved vitality, as well as spirituality [15]. The Motivational Interviewing (MI) approach [14] was also adapted into the program to facilitate behavioral changes [16].

Primarily, the program aims to enhance active ageing for older people in a nursing home to promote the health and wellbeing (i.e., physical, psychological, social, and spiritual functioning) of these individuals, offering emotional security for them in the later stages of life. It was expected that the program could be a valuable option for both the older people and the agencies involved in providing care for them. This study was therefore done with the following objectives: (1) to investigate the concept of active ageing of older people in a nursing home setting. (2) To investigate the effects of an integrative counselling program to promote active ageing for older people in a nursing home. Given the small sample size of 16, this study was done as a pilot study.

## Methods

**Fig. 1:**
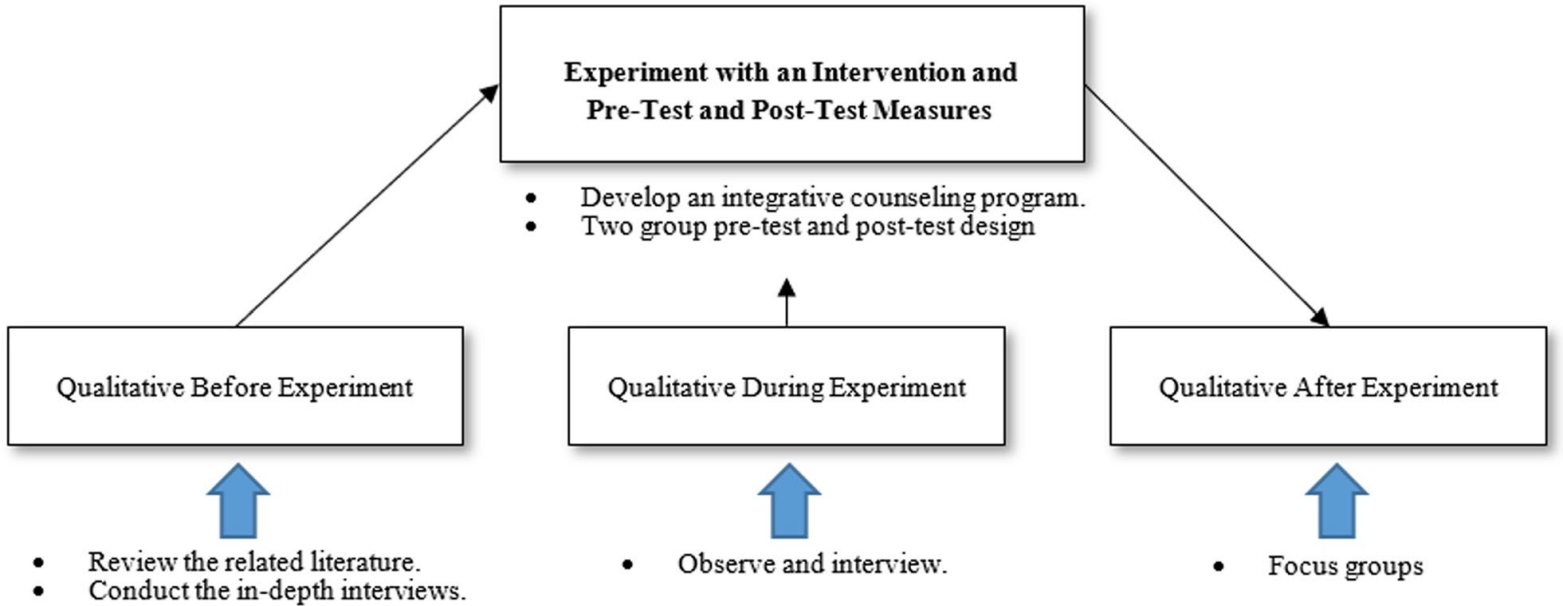
Intervention mixed methods design model

This study was approached on the basis of a pragmatic paradigm in terms of focusing on the realistic application of research to a real-world setting. The Intervention Mixed Methods Design [17] is an approach in which the researcher embeds the qualitative strand before, during and after the quantitative experiment or intervention to promote active ageing for Thai elderly in a nursing home through integrative counselling. This study applied this design and consisted of two phases. Phase 1, the qualitative study involved an investigation of the active ageing concept based on literature reviews and data obtained from the in-depth interviews; the information was used to develop the counselling program. For the quantitative section of phase 2, a pretest–posttest control group design was conducted to compare the post-test results of the experimental and the control groups. For the qualitative section, interviews and observations were conducted during the program; the focus group discussions were conducted after the program (see Fig. 1).

### Phase 1: an investigation of the concept of active ageing based on the context of older people in a nursing home

In this phase, the qualitative method was used. The researcher reviewed the related literature, then selected the participants, which consisted of older people and their caregivers. In-depth interviews were conducted to obtain information that would be used to develop an integrative counselling program.

### Participants

The researcher used the purposive sampling technique to obtain key informants with sufficient information. Based on the criteria, the participants were divided into two groups:

1. Three caregivers of older people with experiences in providing care for older people in a nursing home for more than 3 years.
2. Two older people aged 60 and over that were selected through a screening questionnaire that was developed from Thanakwang’s concept of active ageing [18], this will be described in detail under research instruments. This questionnaire was completed by their caregivers respectively using the seven-item active ageing checklist. The participants who met the criteria were those who answered ‘yes’ in all seven items.

**Fig. 2:**
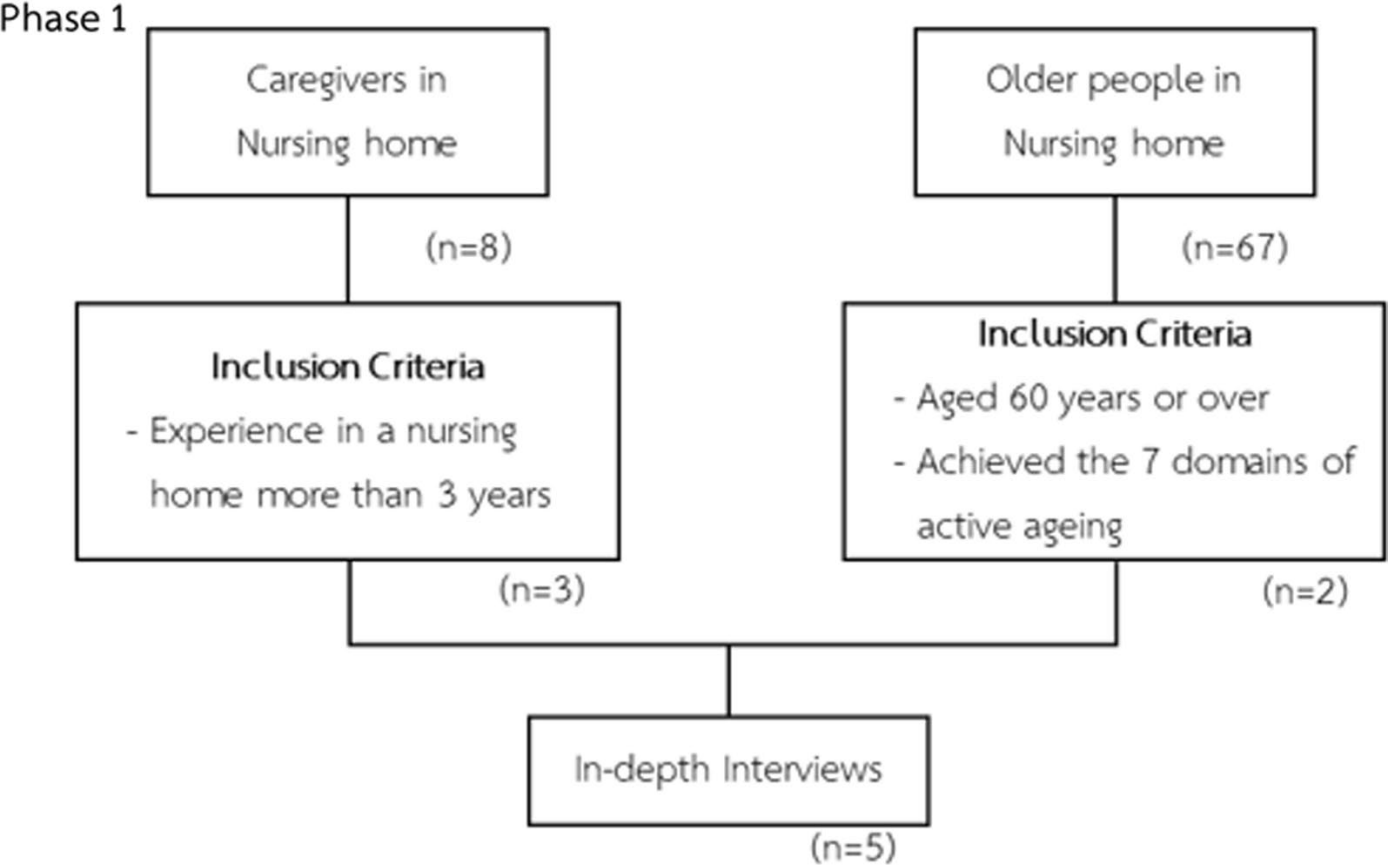
Eligibility criteria and flow chart for phase 1

The eligibility criteria for the participants of phase 1 can be found in Fig. 2.

### Research instruments

(1) The screening questionnaire utilises the seven domains of active ageing from Thanakwang [18] and consists of seven dichotomous questions. Thanakwang’s concept of active ageing was developed from a study that was conducted in line with the WHO’s definition of active ageing on the Thai elderly population in 4 different regions of Thailand which concluded on 7 domains of active aging. Each question corresponds to a domain of active ageing: (1) Being self-reliant—They are self-reliant and are able to go about their daily activities independently, (2) Being actively engaged with society—They actively participate in communal and recreational activities conducted by the nursing home, (3) Growing spiritual wisdom—They uphold strong religious beliefs and live in accordance to religious teachings, (4) Building up financial security—They have financial planning with adequate preparation into their late life, (5) Maintaining a healthy lifestyle—They take matters into their own hands when it comes to maintaining a healthy lifestyle, such as regularly exercising and having a healthy diet, (6) Actively learning—They are constantly interested in engaging in new activities and experiences, such as seeking new ways to improve their health or learning about the latest technology, (7) Strengthening family ties for being cared for in the late life—They are exemplary to their children in strengthening the family bond.

2). A semi-structure in-depth interview was used with 12 open-ended questions, incorporating the seven domains of Thanakwang’s active ageing [18]. Some examples of these questions include: “How do you take care of your health? Is there anything particular that you do?”, “How often do you engage in social activities?”

### Data collection

The researcher made an appointment with the participants to clarify the objectives and the details of the study. The participants had signed the informed consent forms prior to having attended the individual in-depth interview which also includes the consent for the recording of their interviews. Data collected here are mainly observational and through in-depth interviews that were recorded auditorily with 2 different tape recorders. The researcher used recordings of the interview and observations that were noted down during the interview to identify whether the participant’s mood and affect were congruent. Non-verbal cues like smiling, laughing, crying or any other form of emotional expression was noted down during the interview. Recordings and all data collected were confidentially kept under a password protected folder.

### Data analysis

The researcher replayed the tape records of the interviews and compared them to any notes taken for any emotions or expressions from the participants to have a more thorough interpretation of the congruence of mood to affect. The content analysis method (Deductive and inductive analysis) was used to analyze the data. The analyzed data was coded and categorized while themes representing active ageing were developed. This was used to build on and customize the integrative counselling program.

Credibility and reliability of the data was enhanced through peer checking and investigator triangulation. 2 expert supervisors conducted the peer checking and came to a consensus regarding the means of which the data were analyzed and interpreted.

### Phase 2: an investigation of the effects of the integrative counselling program to promote active ageing for older people

A comparative quantitative study was performed to compare the differences of active ageing between the post-test results of the experimental and the control groups. This was done after confirming that pre-test results of both groups were similar. Meanwhile, the qualitative assessments including observations, interviews, and focus group discussion were conducted during and after the treatment.

*Population* 65 older people in a nursing home for the elderly in Samut Prakan province.

**Fig. 3:**
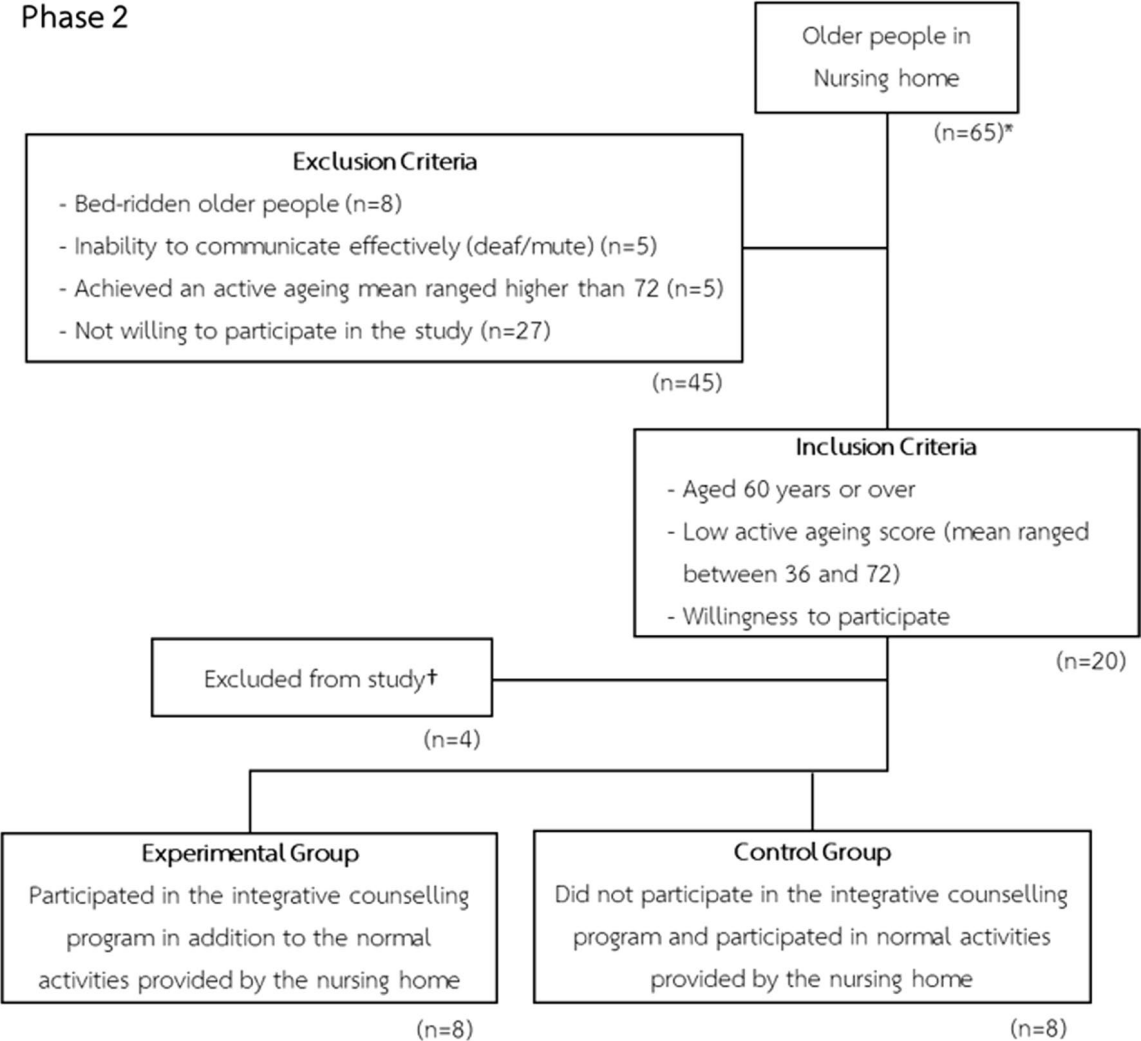
Eligibility criteria and flow chart for phase 2

*Participants* 16 older people. The social demographics of the participants can be found in Table 1. The purposive sampling technique was used to select the participants to take part in the study to select the 16 participants from the population of 65. Inclusion criteria was established as residents aged 60 or over with low active ageing score (mean ranged between 36 and 72) who were willing to participate in the study. The eligibility criteria for the participants of phase 2 can be found in Fig. 3. Cognitive function of the participants were not formally assessed as they were able to go about their daily activities with no problems, there were also no issues regarding neurodegenerative disorders or cognitive problems raised on all of the participants yearly check-up appointments as well. However, it was ensured that all participants were oriented to place, time and person. The simple random assignment technique was used to assign the participants into the experimental and control groups. During the 5 months’ study, both groups engaged normally in recreational activities or any other activities provided by the nursing home, with the only differentiating factor being their participation in the integrative counselling program. A typical day would consist of 3 hearty meals provided by the nursing home, older people in the nursing home would exercise twice a day, they also pray twice daily, once at 0800 and once at 1600. Recreational activities include singing, dancing, having bingo night, this is usually done on the weekends or during public holidays. Furthermore, there are voluntary chores which the older people of the nursing home can partake under their discretion, such as clean certain areas of the home, paint murals, plant trees etc. Every elderly individual has their respective annual check-up doctor’s appointments in addition to any other appointments according to each individual’s own medical or mental health issues.

**Table 1:** Socio-demographic features of the studied population

	Experimental group	Control group
Gender		
Male	4	5
Female	4	3
Age (years)		
60–69	5	4
Over 70	3	4
Marital status		
Unmarried	2	3
Divorced	5	2
Widowed	1	3
Educational level		
Did not received any formal education	2	1
Education up to secondary school	4	3
Education up to college	1	2
Education up to university	1	2
Income		
500–1000 THB	3	4
1001–5000 THB	3	2
> 5000 THB	2	2
Co-morbidities		
None	1	2
Diabetes//Hypertension//Cardiac disease	5	4
Others (Gout—1, Eczema—1, Asthma—1, Kidney Disease—1)	2	2

### Research instruments

Active ageing scale. The Active Ageing Scale—Thailand (AAS-Thai) was used in this study and this scale was developed by Thanakwang [18]. It is a 36-item with 4-point rating scale (giving a total score of 144), developed based on the context of Thai culture, and incorporates the seven domains of active ageing. The following criteria is the interpretation of the active ageing score: a mean score ranging from 36 to 72 represents a low level of active ageing, 73 to 108 represents a medium level of active ageing, and 109 to 144 represents a high level of active ageing. Reliability testing was conducted applying the pilot test with the sample of 200 older people who had similar characteristics to the study participants. The 200 older people were recruited from a nursing home, they had an age range of 60–89 (whereby 22.5% comprised of individuals aged 60–69, 48.5% for individuals aged 70–79, and 30% of individuals aged above 80). Mean age was 75.37 years with standard deviation of 7.68 years. Out of 200, 69% were female. 77% of individuals in the sample pilot test had co-existing co-morbidities, whereas 23% were healthy with no diagnosed medical conditions [19]. The Cronbach’s Alpha of 0.98 was found (see Table 2). The researcher conducted a confirmatory factor analysis (CFA), with first order results showing that all indices met the fit indices criteria (${x}^{2}\hspace{0.17em}$= 377.57, df = 424, ${x}^{2}$/df = 0.89, *p* value = 0.95, CFI = 1.00, GFI = 0.94, AGFI = 0.91, RMR = 0.03, SRMR = 0.03 and RMSEA = 0.00), factor loading ranged from 0.66 TO 0.94, *p* = 0.01. Results of second order CFA also showed that all indices meet the fit indices criteria (${x}^{2}$= 379.38, df = 422, ${x}^{2}$/df = 0.89, *p* value = 0.93, CFI = 1.00, GFI = 0.94, AGFI = 0.91, RMR = 0.03, SRMR = 0.03 and RMSEA = 0.00), factor loading ranged from 0.72 to 0.97, *p* = 0.01 [19].

**Table 2:** Alpha coefficient values of the active ageing scale (AAS-Thai) (n = 200)

Scale	Number of items	Cronbach’s alpha
1. Being self-reliant	8	0.98
2. Being actively engaged with society	8	0.94
3. Growing spiritual wisdom	5	0.90
4. Building financial security	4	0.85
5. Maintaining healthy lifestyle	5	0.89
6. Engaging in active learning	4	0.87
7. Strengthening family ties for being cared for in the late life	2	0.90
Total	36	0.98

2. The integrative counselling program was developed by utilising the results from the in-depth interviews in phase 1 with supplementary psychological theory application from the Satir Model and Motivational Interviewing techniques [13, 14]. In addition, the concept of active ageing in the Thai elderly was studied in great detail to be applied in the program as well. The Satir Model was primarily applied in the program; meanwhile MI techniques were used supplementary to the Satir Model to encourage participants to change their behaviour for the better. Satir positively regards each individual to be unique and to have potential within themselves. Her treatment aims to allow the individual to appreciate the same values that Satir sees in them. These values are deeply rooted to one’s self-esteem or self-worth thus she focused her treatment mainly on enhancing these aspects through the development of self. These principles were applied when conducting this study in the older people of the nursing home. MI is defined as a “client-centred, directive method for enhancing intrinsic motivation to change by exploring and resolving ambivalence” as stated by Miller & Rollnick [14]. Additionally, the FRAMES (Feedback, Responsibility, Advice, Menu Options, Empathy and Self-Efficacy) guide used in MI was also applied in this study, as well as the Stages of Change concept, to allow for the encouragement of participants to express a Self-Motivational Statement (SMS). To supplement this, the researcher underwent a workshop pertaining to application of the Satir Model by Dr. Laura S. Dodson & Miss Maureen Graves, in addition to practical training over a total of 200 h that also utilized the Satir Model in addition to MI techniques.

The integrative counselling program consisted of eight sessions of group counselling preceded by one session of individual counselling, with the objective of allowing the participants to become more familiar with the researcher prior to the start of group counselling. The older people in the experimental group attended 9 counselling sessions, amounting to approximately 16 h per person. The individual counselling sessions are not detailed below as they were conducted according to the needs of each individual participant. However, the group counselling sessions which consisted of 9 individuals (8 participants of the experimental group and the group facilitator, this term is interchangeably used with “researcher” as both are the same person) are elaborated as follows:

*Session 1* Relationship establishment; Inaugurating the development of rapport between the researcher and participants as well as among participants themselves to encourage the older people to actively engage in the organised activities. Activities in this session emphasized the importance of being self-reliant (domain 1) with application of the Satir Model.

*Session 2* Open-mind stage; This session involved sharing of positive experiences and encouraging each other to form the self realisation that even at later stages in life, one can still learn new things. Activities aimed to enhance the engagement to active learning of the participants (domain 6) with application of the Satir Model.

*Sessions 3 and 4* Behaviors modification stage; In these sessions, MI techniques were applied for health behaviour modifications in the participants. Activities were aimed towards maintaining a healthy lifestyle (domain 5) and involved the researcher attempting to change any behaviours regarded as unhealthy with “homework” given during the first session and the monitoring of progress in the later session. 

*Session 5* Self-esteem building; In this session, participants were instructed to design their own drawstring bags with the main focus being on the participants’ active engagement with society (domain 2) being applied in this setting. The Satir Model was used here to guide participants in seeing the resourcefulness within themselves.

*Session 6* Gaining wisdom; Videos of individuals enlightened by Buddhism especially on the views of death and how said individuals got through their later lives with the added spiritual wisdom were shown to the participants, and through the Satir Model and MI techniques, the researcher was able to build spiritual wisdom (domain 3) in the participants through this session.

*Session 7* Realization of yearnings; One of the titles (Yearning) from the “Personal Iceberg” from the Satir Model was utilised in the activities by helping the participants realize their yearnings in the different aspects of life, which allows them to strengthen family ties (domain 7). Additionally, allowing participants to have a sense of reassurance in life aiming towards building up financial security in participants (domain 4).

*Session 8* Farewell activity; The researcher provided participants with a bouquet each, instructing them to offer it to anyone within the group counselling session that they were touched by along with any positive words of affirmation. The Satir Model was used to allow participants to realise the appreciation they have for one another. At the end of the session, the researcher also gifted each participant a bouquet as a means of appreciation and farewell.

3. The interview and observation form. Forms were provided at the end of each session to assess the experimental group participants’ understanding and opinion on the session conducted that day along with any comments they were willing to provide.

4. The focus group discussion was proven to be a reliable method of collecting data according to Sakunpong et al. [20] and therefore was included after the program, as a tool to get to know the experimental group’s opinions on the intervention used in this study. Examples of some questions in the discussion include: (1) Questions in relation to positive changes resulted from active ageing: “How did the program influence your daily routine?”, “Did the program positively influence how you took care of yourself in comparison to before joining?”, “Are there any changes in your stance towards the feeling of being secure in life while ageing?” (2) Questions in relation to participants’ feelings towards the activities, group facilitator, and group atmosphere: “How did you feel about the activities, group facilitator, and the surroundings?” (3) Questions with regards to the participants’ suggestion on the program arrangement: “Overall, how did you feel about the program conducted?” “Are there any suggestions you would want to propose to further better the program?”

### Data collection

The researcher clarified the objectives of the study, research procedures and precautions taken to protect the confidentiality of the participants’ information, after which the participants signed the informed consent forms which included consent for recording of the interviews. Pre-test and post-test active ageing in the participants were measured using AAS—Thai mentioned above. The type of data recorded was primarily observational and through focus group discussions that were recorded auditorily and visually with 2 different tape recorders and 1 video recorder. For this phase, the researcher assistant aided in noting down non-verbal cues and any form of expression from the participants as the researcher was the facilitator for the focus group discussion. Recordings and data collected were confidentially kept under a password protected folder.

### Data analysis

For the quantitative data, the normality of the data was tested with the Shapiro–Wilk test and was concluded to be a non-normal distribution of data, which was why non-parametric statistics were used. (Shapiro Wilk test during pre-test: 0.467, sig. = 0.000; post-test: 0.817, sig. = 0.004) Nonparametric statistics, namely the Mann–Whitney U-Test, was used to test the differences of pre-test and post-test active ageing scores between the experimental and control groups. As for the qualitative data, the researcher replayed the tape records of the focus group discussion and reviewed the notes taken for comparison to obtain a more thorough interpretation of congruence of mood and affect. The content analysis method (Deductive and inductive analysis) was performed to analyze the qualitative data which were obtained from the interviews and the focus group discussion. The analyzed data was coded, categorized and interpreted into themes and corresponding sub-themes. This was used to draw a conclusion on the factors promoting active ageing in the older people.

Peer checking and investigator triangulation were used to enhance the credibility and reliability of the data. Peer checking was conducted by 2 expert supervisors and came to a consensus on the manner which the data was collected and interpreted.

## Results

### Phase 1: active ageing concept based on the context of older people in a nursing home

The results of phase 1 consisted of a qualitative data analysis which fulfils objective 1 listed earlier in the paper:

An overarching major theme was found to be the developmental processes of the participants leaning towards a gradual application of active ageing in their daily routines with 4 sub-themes that are detailed as below:

(1) Health development referred to promotion of the health status of older people. Also, this dimension included processes by which older people realized the importance of maintaining their health with a combination of exercise, healthy diet and being compliant to their regular check-up appointments.

They (the older people) take matters regarding their health into their own hands, you can always see them trying to be regularly active with some of them following physiotherapy exercises or doing simple arm swinging exercises in the early hours of the day. I’ve always reminded them that ‘Money can’t buy health’ in the hopes to motivate them to push themselves further. (Fah, caregiver).

(2) Spiritual development referred to the process in which older people performed religious practices and regularly exercised their faith in Buddhism. As a result, older people were able to let go of any negativity as well as harness their spiritual energy which contributed towards their spiritual development and general happiness.

I regularly pray twice a day at the nursing home. In addition, I would routinely participate in almsgiving at the temple. I believe that the older you get, the more you rely on spirituality and that regular practice is essential. (Dio, one of the older people in the nursing home).

(3) Active engagement referred to the growth in the willingness of older people to engage in activities of the nursing home as well as any active social participation. This includes any recreational activities that were held to improve their physical and mental health, self-esteem and to create a sense of belonging amongst older people.

Several groups of students have organised activities here and the older people really enjoyed doing them. There were a variety of tasks within the activities whereby the older people would split into groups to participate. Some of the students prepared games such as ring toss or charades for them (the older people). The activities aimed to create a harmonious atmosphere among the older people as well as to further enhance their creativity. (Pla, caregiver).

(4) Psychosocial support referred to the gradual acquisition of emotional support and encouragement in older people. This significantly increased the awareness of their self-worth and led to positive impacts psychologically whereby they had reduced feelings of anxiety with regards to their later life.

The love and support given to the older people are not limited to us (caregivers), but also by the volunteers that visit. Occasionally, birthday surprises are organized for them (the older people), this had deeply touched one of our elders to tears, where she mentioned that no one has done this for her before. Another older person told me ‘I feel a sense of security here, I know that when I take my last breath, the nursing home would take care of my funeral’. The older people here constantly mention that their time here feels worthwhile and that they’ve always felt appreciated unlike the situation at their own respective homes. (Fah, caregiver).

### Phase 2: the effects of the integrative counselling program to promote active ageing for older people in a nursing home

The results of the quantitative data analysis in phase 2 which achieves objective 2 as mentioned above.

### Quantitative results

Nonparametric statistics, the Mann–Whitney U-Test was used to analyze the quantitative data. Prior to the study, a pre-test was done to confirm that there were no discrepancies between the active ageing scores of the experimental and control group. The differences between post-test active ageing scores of the experimental and control groups were determined. The results revealed that the experimental group had a higher overall active ageing score in comparison to the control group (*p* < 0.05). Additionally, each individual domain from the active ageing scale also proved to have statistically significant results in the experimental group with a *p* value of < 0.05. (Table 3).

**Table 3:** Comparisons of active ageing of the results between the experimental and control

Domains of active ageing / period	Experimental group		Control Group		Z Mann–Whitney U
	Mean rank	Sum of rank	Mean rank	Sum of rank	
1. Being self-reliance					
Pre-test	8.63	69.00	8.38	67.00	0.11
Post-test	12.50	100.00	4.50	36.00	3.38*
2. Being actively engaged with society					
Pre-test	8.63	69.00	8.38	67.00	0.11
Post-test	12.06	96.50	4.94	39.50	3.01*
3. Growing spiritual wisdom					
Pre-test	6.75	54.00	10.25	82.00	1.50
Post-test	11.25	90.00	5.75	46.00	2.36*
4. Building up financial security					
Pre-test	8.31	66.50	8.69	69.50	0.16
Post-test	10.81	86.50	6.19	49.50	2.00*
5. Maintaining healthy lifestyle					
Pre-test	8.75	70.00	8.25	66.00	0.22
Post-test	11.88	95.00	5.13	41.00	2.93*
6. Engaging in active learning					
Pre-test	8.31	66.50	8.69	69.50	0.17
Post-test	10.88	87.00	6.13	49.00	2.15*
7. Strengthening family ties for being cared for in the late life					
Pre-test	8.00	64.00	9.00	72.00	0.49
Post-test	10.81	86.50	6.19	49.50	2.04*
Active ageing (overall)					
Pre-test	7.31	58.50	9.69	77.50	1.01
Post-test	12.50	100.00	4.50	36.00	3.37*

*p < 0.05

### Qualitative results

Qualitative results were obtained from interviews conducted during the study as well as from the focus group discussion with the overall results supporting the quantitative results. The results include the factors promoting active ageing in older people which are described below:

1. Activities referred to the counselling psychology program that enabled the participants of the experimental arm to acknowledge their capabilities, gain self-esteem, adapt better, and be able to cope with life’s challenges. The positive interactions within the program allowed the participants to get better acquainted and learn from the experience of others which increased their interpersonal bond.

I spent my whole life alone, with no partner or parents, until today. At one point, I even thought of ending my own life, but decided otherwise as it was considered a sin according to Buddhist beliefs. After participating in this program, and through the various activities organized, I came to value myself much more, I’ve come to realise that I too, am worthy of the love and care of others. (Yim, participant in the experimental group)

2. Group facilitator referred to a skillful, encouraging and knowledgeable counselling psychologist with the following characteristics: having an approachable personality, being attentive to the participants’ opinions and being able to communicate with participants comfortably, open mindedly and respectfully. These characteristics assisted in harnessing the participants’ confidence and self-esteem, leading to increased trust between the participant and the facilitator.

Since you (group facilitator) came, you’ve brightened my dull days. I feel flattered that you are willing to understand old people like us and have the patience to listen to our long-winded stories. I really appreciate your kind and pleasant personality, you’ve always been very polite and communicated with us with such warmth. (Jack, participant in experimental group)

3. Group atmosphere referred to the interactions among everyone in the group counselling session (participants and facilitator) which led to a closeness within the group that was not present at the start, in addition to the physical surroundings that increased the effectiveness of activities, such as sitting comfortably in a sheltered room.

The atmosphere in this room is much better than the outside, it is peaceful and quiet, which helps us focus better on the activities at hand. The electric fan enables us to have a comfortable temperature. Even though we have been in the same nursing home for quite some time, I am glad that all of us have gotten to know each other better through this program. (Glom, participant in experimental group)

## Discussion

This study was conducted to investigate active ageing in older people in the setting of a nursing home and to promote active ageing through an integrative counselling program by way of an intervention mixed methods design. The study was conducted in 2 phases, phase 1 investigated the concept of active ageing based on the viewpoints provided by the older people and caregivers in the nursing home, an integrative counselling program was developed based on phase 1 to be applied in phase 2. Phase 2 involved investigating the effects of the integrative counselling program to promote active ageing in older people in the same nursing home. Both phases provided overarching themes that are discussed below.

### Phase 1: the concept of active ageing based on the context of older people in a nursing home

The process of developing the participants’ gradual application of active ageing in their daily routines in a Thai nursing home for older people comprises 4 sub-themes as detailed in the results section. The sub-themes are consistent with the WHO’s concept of active ageing [21] that consists of 3 pillars including health, participation and security. Firstly, “Health development” is in line with the health aspect, in which older people prioritise the maintenance of their physical health by changing their everyday routines and behaviors to lean towards an active and healthy lifestyle. According to Thanakwang [18], keeping yourself busy and maintaining a healthy lifestyle closely relates to an individual’s physical and psychological health. Next, “Active engagement” and “Spiritual development” is congruent with participation, where older people actively seek to expand their spiritual wisdom through prayers or almsgiving, and also proactively participate in recreational and social activities organised. Experiencing spiritual growth can be considered an aspect of active ageing, as well as a core aspect of having a meaningful life that includes meaningful relationships with others, including God [18, 22]. Moreover, the spirituality of the Thai elderly is related to both intra-personal and inter-personal relationships that are deeply entrenched with religion, particularly Buddhism as it is unique to Thai culture in which most uphold the same beliefs [18, 23]. Additionally, a crucial indicator of healthy and active ageing includes being able to keep oneself actively engaged with any enjoyable or meaningful activities [24–26]. Furthermore, it is said that older people who actively engage themselves in volunteering or charitable activities are also more likely to exhibit qualities of active ageing [27]. Lastly, “Psychosocial support” corresponds to security, whereby older people have acquired the emotional support and encouragement from the people around them in addition to a sense of security in their current position and physical surroundings. With us living in a dynamically developing world with rapid changes in socioeconomics and family structures, it is incredibly crucial that older adults should live in the absence of any abusive or threatening situations and the presence of financial security, this greatly enhances one’s sense of security and autonomy [24, 28, 29]. Elderly individuals that actively maintain their health, engage in social activities, have a high spirituality as well as adequate social support contribute to the overall wellbeing of the individual and are also the basic constitutions of active ageing [30].

### Phase 2: the effects of the integrative counselling program to promote active ageing for older people

This study was conducted as a pilot experiment as there was a small sample size of 16 participants. According to the results of the quantitative study, it was found that participants of the experimental group had a higher overall active ageing score than the participants of the control group (*p* < 0.05). This is due to the fact that older people engaging in the program have actively participated in improving themselves by changing their thoughts and behaviours to optimize their mindset to be in line with the qualities of an older person with active ageing. The application of MI techniques supplementary to the Satir Model which are adapted into the program used in this study. This emphasizes on the acknowledgement of one’s values, as well as the boosting of one’s self-esteem and realization of one’s self worth and also further facilitating positive behaviour change in older people. Moreover, the participants in the experimental arm have gained insight with regards to their self-worth, and have learnt to accept themselves for who they are, creating a different level of inner peace. The program also allowed the participants to be able to have confidence in themselves with a higher self-esteem to adjust and deal with their current reality along with any challenges or difficulties that may come their way. Additionally, the participants were able to enhance their spiritual wisdom which also contributed to a stronger vitality towards their lives [31].

The qualitative data analysis conducted in phase 2 through the focus groups and interviews consisted of 3 aspects: activities, group facilitator and group atmosphere. The activities allowed the participants to realise and acknowledge their strengths which led to a more valuable view of oneself. These activities were adapted by the researcher based on information obtained from the qualitative study in phase 1 as well as existing concepts of active ageing to be able to customize the program for the selected audience. Furthermore, the researcher developed the program based on Thanakwang’s concept of active ageing [18] which incorporates positive qualities of older people in the context of Thai culture. Additionally, the researcher also applied the Satir Model which strongly emphasizes on the acknowledgement of one’s values, as well as the boosting of one’s self-esteem and realization of one’s self-worth [15]. MI techniques have also been used supplementary to the Satir Model which facilitates positive behaviour change in older people. The combination of this had led to older people being able to cope with challenges and adjust to difficult situations calmly and competently [16, 32, 33].

The role of the group facilitator in group counselling is essential for a group’s progress towards a certain common goal. In this case, for participants to be able to develop active ageing, the facilitator not only guides the direction that the group is heading but also provides any psychological counselling and support when needed. According to [34], it is the counselling psychologist/facilitator’s responsibility to enable their clients to see the good within themselves, to further enhance the awareness of their self-worth, and to understand that a healthy sense of self-worth also corresponds to a higher spirituality. According to Satir [35], it is crucial that counselling psychologists or the group facilitator in the case of group counselling are aware of their own attitudes and treatment towards clients. The facilitator is expected to create an atmosphere that is open and trusting to enable the participants to be able to open up and share their thoughts and feelings. In the words of Corey et al. [36], older people are individuals that are highly vulnerable and sensitive. With that being said it is of utmost importance that the psychologist/facilitator treats older people with care and respect as older people require feelings of acceptance, understanding, and warmth. Group atmosphere has proved to be an important feat of group counselling, which has been proven by a study conducted by Der Pan [37], who also utilised the Satir Model and found that group atmosphere played a significant role in the efficacy of counselling conducted and in the case of this study, further improved the influence the activities had on the participants which was shown in the results. One study showed that the atmosphere, location and time of day of group counselling is a crucial factor for obtaining better discussions. y[7] A good location for counselling would be a comfortable, personable and quiet enclosed space as which ensures the preservation of confidentiality. Additionally, a better atmosphere led to a conducive environment where the participants were at liberty to share their experiences with each other and have discussions working through problems together.

### Limitations and directions for future research

A key limitation of this study was its sample size. There was limited area of coverage as only one nursing home was studied. Further research can be done with the same counselling program but with an increased sample size. Furthermore, conducting the program with other groups of people (individuals with different co-morbidities) that stay in nursing homes in different areas. A repeat study can also be conducted a few months after this study to further analyze the long-term effects of the counselling program. Other factors that promote active ageing can also be analyzed in future research as well, such as family bonding or ability of older people to cope with problems.

## Conclusion

Individuals involved with the provision of care for older people in nursing homes in both public and private sectors such as older people caregivers, psychologists etc. are also able to apply the 4 sub-themes from phase 1 to encourage development of active ageing in older people. The integrative counselling program in this study has been customized for the Thai elderly in a nursing home setting and can be utilised by psychologists to enhance active ageing in older people in similar settings. It is notable that the older people engaging with this program are able to be independent, preserve their mental and physical capacity, have a high self-esteem, become better adjusted to any adverse events in their current reality and also are better able to realise their self-worth. This program is also designed to improve the spirituality and vitality of older people, which is especially relevant to the characteristics of Thai elderly who consider religious principles as their spiritual anchor. Religious practices such as going to a temple or almsgiving enable older people to feel less distressed and more mindful, which better prepares them for the later stages of life yet to come.

## Data Availability

Datasets generated and/or analysed during the current study are not publicly available because permission was not obtained from the participants to share their data publicly but could be available from the corresponding author on reasonable request.

## Abbreviations

CFA: Confirmatory Factor Analysis
FRAMES: Feedback, Responsibility, Advice, Menu Options, Empathy and Self Efficacy
MI: Motivational Interview
SPSS: Statistical Package for Social Sciences
WHO: World Health Organization
